# The relative age effect among Chinese junior men’s tennis players and its impact on sports performance

**DOI:** 10.1371/journal.pone.0292443

**Published:** 2023-10-10

**Authors:** Yisheng Aku, Cheng-bo Yang

**Affiliations:** Department of Athletic Training, Chengdu Sport University, Chengdu, China; Instituto Politécnico de Santarém: Instituto Politecnico de Santarem, PORTUGAL

## Abstract

The relative age effect (RAE) has been the subject of many studies, but no relevant literature has discussed the phenomenon of RAE in Chinese tennis. Numerous studies have demonstrated that RAE significantly contributes to brain drain and other occurrences that create inequity. This paper analyzes the birth dates and year-end rankings of all male players (N = 2697) who participated in China’s junior tennis tournaments (U12, U14, U16) between 2014 and 2019 and who were selected for China’s National Junior Team in 2019 and 2020; the paper classifies the birth dates into quarters and semesters. One of the research objectives of this study is to analyze whether RAE exists in Chinese junior men’s tennis and whether RAE exerts an effect on athletes’ performance. Differences between the observed and expected birthdate distributions were tested using chi-square statistics, and subsequent calculations were tested using odds ratios. The study found that RAE was present in all Chinese junior male tennis sports groups (p<0.001). The percentages of athletes born in the first half of the year were 56.4% (U12), 60.4% (U14), and 60.4% (U16), and the percentages of those born in the first quarter were 34.1% (U12), 36.4% (U14), and 37.1% (U16). Athletes with birth dates closer to the beginning of the year had a higher probability of achieving excellent athletic performance as a result of RAE, whereas those who were born near the end of the year had a more difficult time achieving strong athletic performance.

## Introduction

“Relative age” refers to the age difference between different individuals who are in the same group for some form of activity [1]. For example, in China, children who were born between September 1 and August 31 of the following year are eligible to attend primary school. Due to their varied birth dates, students in the same grade can have age gaps of up to one year. This age discrepancy is referred to as “relative age” [2], and the “relative age effect” (RAE) is a developmental problem that athletes, coaches, and policymakers face in sports [3, 4]; RAE refers to the deviation of the distribution of birthdates of selected athletes from the normal distribution in the population [5]. It occurs when the proportion of relatively older people in a certain activity group is higher. Although this phenomenon affects all team sports, it impacts the two sexes differently [6–8]. Since boys attain their peak height velocity nearly two years later than girls, girls undergo puberty earlier [9].

According to sports science research, RAE makes athletes born in the selection year or early in the competition year more mature in terms of their physical, emotional, cognitive, and performance advantages than athletes born later in the same year [10–12]. This renders them more likely to be selected into higher-level sports teams and talent training programs [1, 6]. Additionally, the unequal allocation of resources to coaches and training facilities can exacerbate the RAE. This might result in bias in favor of people who are physically more advanced than others, thereby creating squandered opportunities [13, 14]. Furthermore, this could cause players to fully abandon their sport [15–17].

The impact of RAE has been researched in relation to sports [1–4], education systems [18–20], specific medical diagnoses [21, 22], and cognitive tasks [23]. The first RAE research was reported in the field of education [24]; Grondin et al. [25] were the first to publish results identifying RAE in sports after noticing an excess of relatively older players among male and female volleyball and ice hockey players at the recreational, competitive, and senior professional levels in Canada.

Numerous studies regarding the impact of the RAE have been published in the past ten years, including those concerning soccer [6, 10, 13, 26], basketball [27, 28], water polo [29], volleyball [30, 31], ice hockey [32], rugby [29], and handball [33]. Because research has demonstrated that RAE can exert various negative effects in sports, numerous discussions about RAE triggering mechanisms in sports have gradually emerged; specifically, the triggers are multifactorial and mixed, depending upon factors such as age group and position in the game [34], socio-cultural context [35], level of competition [27], and historical events [6]. This has resulted in the relative age effect becoming one of the most prominent and influential factors in sports, as it may contribute to talent selection and identification bias [36–38]. Although there have been studies concerning RAE mitigation programs from different perspectives, no relevant studies have been conducted in China, and very few studies have truly implemented the relevant measures. Based on the aforementioned literature search, it is evident that while the RAE is ardently contested in team sports such as soccer, ice hockey, and basketball, there are significantly fewer studies regarding the subject in relation to individual sports, such as tennis, swimming, table tennis, combat sports, gymnastics, and swimming.

In tennis, RAE has been uncovered in both young players and elite athletes. The skewed birthdate distribution of 12-to-16-year-old tennis players in the Dutch youth league rankings was initially discovered by Dudink et al. [39]. Giacomini [40] found that the relationship between young male tennis players’ birth dates and sports success gradually decreased with age; conversely, among female players, birth dates exerted no effect on tennis ranking.

Based on various studies regarding elite tennis players, Edgar, O’ Donoghue [41] found that 58.9% of both male and female tennis players who had achieved the highest professional level were born in the first six months of the year. They also noted that there was a notable birth season skew for both women’s singles and men’s singles participants in the tennis Grand Slam tournaments of 2002 and 2003. In the context of RAE, Florian Loffing and Schorer [42] conducted a survey of the top 500 male professional tennis players in the ATP year-end rankings between 2000 and 2006. The RAE was clearly present when only right-handed athletes were taken into account. The left-handed tennis players, on the other hand, exhibited no signs of RAE.

Studies have also specifically examined young tennis players. Pacharoni et al. [43] analyzed different groups of teenage tennis players and professional players in Brazil and found that most U12-U18 players (65.2%) were born in the first half of the year; additionally, their birth dates and expected had a significantly skewed distribution. Among professional athletes, the distribution of birth dates was relatively even. According to Agricola et al.’s findings [44], most of the elite junior athletes who competed in the WJTF from 2007 to 2011 were born in the first quarter of the year (38.9%), while only 10.9% were born in the last quarter. Gerdin et al. [45] examined the RAE among Swedish tennis players born between 1998 and 2001. All of the top-ranked athletes displayed a moderate RAE when their birthdates were compared to those of the corresponding Swedish population. On average, 64.1% of the top 10 athletes in each category were born in the first half of the year, and the RAE intensified as athletes’ rankings improved.

The RAE in sports has been the subject of substantial research and discussion, but there is still minimal relevant research in China. A large number of studies have demonstrated that RAE can cause many unfair phenomena, such as talent loss [16, 46, 47]; thus, it is necessary to analyze each sport to determine whether RAE exists and to address the problem at an early stage. The dearth of literature regarding the RAE in Chinese tennis or research regarding the correlation between RAE and athletic performance is detrimental to the cultivation of tennis talent in China and the study of the RAE in sports around the world. In conclusion, more research is required to clarify the current state of knowledge regarding the RAE among young Chinese male tennis players. The primary research objective of this study is to analyze whether the RAE affects all male players (N = 2697) who participated in China’s junior tennis tournaments (U12, U14, U16) from 2014 to 2019 and who were selected for China’s National Junior Team in 2019 and 2020. A further purpose is to study the effects of the RAE in relation to the performance of Chinese junior tennis players and to explore the science of selection indexes in Chinese junior tennis. These indexes can serve as a foundational resource for future studies regarding the RAE, youth sporting events, and talent scouting.

## Methods

### Participants

This study recorded the birth date and year-end ranking results of all the 2590 male athletes who participated in the Chinese junior tennis tournaments (U12, U14, and U16) from 2014 to 2019. The 107 male athletes chosen for China’s National Junior Tennis Team and Junior Reserve Team in 2019 and 2020 were listed with their birth year and year-end ranking.

Rankings and birth months for the research participants was downloaded through the open-access online data-bases provided by the China Tennis Association’s official website (www.Tennissport.org.cn). Data extraction included each player’s name, birthdate, and Year-end ranking results. Due to the study data being in the public domain, no informed consent and approval by an Ethical Committee were required.

### Statistical analysis

Based on the birthdates, each player was assigned to one of four relative age quarters (Q) and two semesters (S) to calculate the RAE of the different age categories [4]. The cut-off date for tennis in China is January 1. Therefore, the year was divided into quarters: Q1 = January, February, and March; Q2 = April, May, and June; Q3 = July, August, and September; and Q4 = October, November, and December. Furthermore, the year was also divided into semesters: S1 = January to June and S2 = July to December for half-year distributions.

To examine RAE, the distribution of the participants’ birth months across Q1–Q4 was examined using Chi-square t (X²) goodness of fit test. The theoretical (expected)frequency distribution was Q1 = 90.25/365.25, Q2 = 91/365.25, Q3 = 92/365.25, Q4 = 92/365.25, which is in relative values: Q1 = 24.71%, Q2 = 24.91%, Q3 = 25.19%, Q4 = 25.19% [41]. The effect size was set at an alpha level of p < 0.05 for statistical significance and p < 0.01 for a highly significant difference. Odds ratios (OR) with 95% confidence intervals (95% CI) were calculated between Q1/Q4 [4]. The OR comparisons were interpreted as follows: OR < 1.22 was negligible, 1.22 ≤ OR < 1.86 was small, 1.86 ≤ OR < 3.00 was medium, and OR ≥ 3.00 was large [48]. All the statistical analyses were performed using SPSS 25.0 (USA). Finally, In order to more clearly compare the relative impact of birth date on the performance of young tennis players, the total number of players in each group was evaluated and compared with the total number of players in the top 30 at the end of the year and the total number of players in the bottom 30 at the end of the year.

## Results

### Relative age effect of the men’s U12 group from 2014 to 2019

As is evident in Table 1, the percentages of athletes born in the first to fourth quarters of the U12 group were 34.1%, 22.3%, 21.7%, and 22.9%, respectively, and the percentages born in the first and second half of the year were 56.4% and 43.6%. The chi-square value (X²) was 49.305, p < 0.01, and the degree of skewness of the athletes’ birth dates displayed a highly significant difference.

**Table 1:** Distribution and analysis of the birth dates of male U12 athletes from 2014–2019.

Year	Classify	Birthdate distribution					Semesters		Data analysis		
		Q1	Q2	Q3	Q4	Total	S1	S2	X²	p	OR
**2014**	n	43	17	30	31	121	60	61	11.198	.011*	1.39
	%	35.5	14.0	24.8	25.6	100.0	49.6	50.4			
**2015**	n	44	27	25	26	122	71	51	8.033	.045*	1.69
	%	36.1	22.1	20.5	21.3	100.0	58.2	41.8			
**2016**	n	39	20	26	21	106	59	47	8.642	.034*	1.86
	%	36.8	18.9	24.5	19.8	100.0	55.7	44.3			
**2017**	n	43	26	18	16	103	69	34	17.583	.001**	2.69
	%	41.7	25.2	17.5	15.5	100.0	66.9	33.1			
**2018**	n	39	27	14	20	100	66	34	13.840	.003**	1.95
	%	39.0	27.0	14.0	20.0	100.0	66.0	34.0			
**2019**	n	165	127	114	136	542	292	250	10.369	.016*	1.21
	%	30.4	23.4	21.0	25.1	100.0	53.8	46.2			
**Total**	n	373	244	227	250	1094	617	477	49.305	.000**	1.49
	%	34.1	22.3	20.7	22.9	100.0	56.4	43.6			

Q1–Q4: The first to the fourth quarter, X²: chi-square value, p > 0.05: no significant difference, p < 0.05: significant difference*, p < 0.01: very significant difference**, OR: ratio of Q1 and Q4.

There is an evident relative age effect among Chinese U12 male tennis players. The birth dates of athletes from 2014 to 2019 exhibited a skewed distribution every year, and there were statistically significant differences (p < 0.05); based on the Q1/Q4 (OR) values, 2019 was the only year with no obvious impact; all other years had different degrees of impact. In general, the date of birth skew of the U12 athletes displayed a statistically significant difference (p < 0.01). Among U12 male tennis players between 2014 and 2019, there was a trend of exacerbation followed by a steady decline in the RAE; overall, there was neither a notable improvement nor an exacerbation.

### Relative age effect of the men’s U14 group from 2014 to 2019

It is evident from Table 2 that the percentage of athletes born in the first to fourth quarters of the U14 group were 36.4%, 24.0%, 17.6%, and 22.1%, respectively, and the percentage born in the first and second half of the year were 60.4% and 39.6%. The chi-square value (X²) was 67.005, p < 0.01, and the degree of skewness of the athletes’ birth dates exhibited a highly significant difference.

**Table 2:** Distribution and analysis of the birth dates of male U14 athletes from 2014–2019.

Year	Classify	Birthdate distribution					Semesters		Data analysis		
		Q1	Q2	Q3	Q4	Total	S1	S2	X²	p	OR
**2014**	n	56	29	16	21	122	85	37	31.246	.000**	2.67
	%	45.9	23.8	13.1	17.2	100.0	69.7	30.3			
**2015**	n	39	11	16	21	87	50	37	20.540	.000**	1.86
	%	44.8	12.6	18.4	24.2	100.0	57.4	42.6			
**2016**	n	33	36	20	32	121	69	52	4.917	.178	1.03
	%	27.3	29.8	16.5	26.4	100.0	57.1	42.9			
**2017**	n	41	27	24	25	117	68	49	6.453	.092	1.64
	%	35.0	23.1	20.5	21.4	100.0	58.1	41.9			
**2018**	n	44	30	19	20	113	74	39	14.327	.002**	2.20
	%	38.9	26.5	16.8	17.7	100.0	65.4	34.6			
**2019**	n	100	73	56	71	300	173	127	13.413	.004**	1.41
	%	33.3	24.3	18.7	23.7	100.0	57.6	42.4			
**Total**	n	313	206	151	190	860	519	341	67.005	.000**	1.65
	%	36.4	24.0	17.6	22.1	100.0	60.4	39.6			

Q1–Q4: The first to the fourth quarter, X²: chi-square value, p > 0.05: no significant difference, p < 0.05: significant difference*, p < 0.01: very significant difference**, OR: ratio of Q1 and Q4.

Among Chinese U14 male tennis players, with the exception of 2016 and 2017, the date of birth skews in other years were statistically significantly different, even for athletes born in the first half of 2016 or 2017; they were also significantly higher than for those born in the second half of the year. Judging from the S1/S4 (OR) value, only the impact of 2016 was negligible, and other years had varying degrees of impact. In terms of the total number of U14 players, the date of birth was more skewed, and the impact on the statistical significance was highly significant (p < 0.01). Therefore, among U14 Chinese U14 male athletes, the RAE was evident. Overall, there was no significant trend of improvement or exacerbation of the RAE among U14 male tennis players from 2014 to 2019.

### Relative age effect of the men’s U16 group from 2014 to 2019

Table 3 indicates that the percentages of athletes born in the first to fourth quarters of the U16 group were 37.1%, 23.3%, 18.6%, and 21.1%, respectively, and the percentages born in the first and second half of the year were 60.4% and 39.6%. The chi-square value (X²) was 52.553, the p-value was < 0.01, and the degree of skewness of the athletes’ birth dates displayed a highly significant difference.

**Table 3:** Distribution and analysis of the birth dates of male U16 athletes from 2014–2019.

Year	Classify	Birthdate distribution					Semesters		Data analysis		
		Q1	Q2	Q3	Q4	Total	S1	S2	X²	p	OR
**2014**	n	41	22	22	20	105	63	42	11.152	.011*	2.05
	%	39.0	21.0	21.0	19.0	100.0	60.0	40.0			
**2015**	n	45	28	14	17	104	73	31	22.692	.000**	2.65
	%	43.3	26.9	13.5	16.3	100.0	70.2	29.8			
**2016**	n	43	25	23	27	118	68	50	8.508	.037*	1.59
	%	36.4	21.2	19.3	22.7	100.0	57.6	42.4			
**2017**	n	21	19	11	20	71	40	31	3.535	.316	1.05
	%	29.6	26.8	15.5	28.2	100.0	56.4	43.6			
**2018**	n	36	25	21	23	105	61	44	5.133	.162	1.5
	%	34.3	23.8	20.0	21.9	100.0	58.1	41.9			
**2019**	n	50	29	27	27	133	79	54	11.331	.010*	1.85
	%	37.6	21.8	20.3	20.3	100.0	59.4	40.6			
**Total**	n	236	148	118	134	636	384	252	52.553	.000**	1.76
	%	37.1	23.3	18.6	21.1	100.0	60.4	39.6			

Q1–Q4: The first to the fourth quarter, X²: chi-square value, p > 0.05: no significant difference, p < 0.05: significant difference*, p < 0.01: very significant difference**, OR: ratio of Q1 and Q4.

For the older group of Chinese junior tennis players (U16), the degree of skewness in the players’ dates of birth was still evident; the years of age were not statistically significant for only 2017 and 2018. In terms of the Q1/Q4 (OR) values, only the impact of 2017 was negligible, and other years had varying degrees of impact. The date of birth skew of the total number exhibited a highly significant difference (p < 0.01), and the RAE was clear among Chinese U16 junior men’s tennis players. Overall, there was no significant regular change in the impact of the RAE among U16 male tennis players between 2014 and 2019.

### The relative age effect within the Chinese national men’s junior tennis team

As is evident in Table 4, the percentages of athletes born in the first to fourth quarters on the Chinese National Men’s Junior Tennis Team (2019–2020) were 44.9%, 20.6%, 15.9%, and 18.7%, respectively, and the percentages born in the first and second half of the year were 65.4% and 34.6%. The chi-square value (X²) was 22.981, the p-value was < 0.01, and the degree of skewness of the athletes’ birth dates displayed a highly significant difference.

**Table 4:** Distribution and analysis of the birth dates of Chinese national men‘s junior tennis team.

Year	Classify	Birthdate distribution					Semesters		Data analysis		
		Q1	Q2	Q3	Q4	Total	S1	S2	X²	p	OR
**2019**	n	17	4	6	9	36	21	15	10.889	.012*	1.89
	%	47.2	11.1	16.7	25.0	100.0	58.3	41.7			
**2020**	n	12	4	3	5	24	16	8	8.333	.040*	2.40
	%	50.0	16.7	12.5	20.8	100.0	66.7	33.3			
**2020 Reserves**	n	19	14	8	6	47	33	14	8.915	.030*	3.17
	%	40.4	29.8	17.0	12.8	100.0	70.2	29.8			
**Total**	n	48	22	17	20	107	70	37	22.981	.000**	2.80
	%	44.9	20.6	15.9	18.7	100.0	65.4	34.6			

Q1–Q4: The first to the fourth quarter, X²: chi-square value, p > 0.05: no significant difference, p < 0.05: significant difference*, p < 0.01: very significant difference**, OR: ratio of Q1 and Q4.

In general, the dates of birth among the Chinese men’s national junior tennis team players were highly skewed, and there was a statistically significant difference in each group (p < 0.05); the overall degree of skewness was highly significant (p < 0.01), with the OR value as high as 2.8 and the proportion of births in the first half of the year as high as 65.4%. The relative age effect is thus evident in the Chinese national men’s junior tennis team.

### The relative age effect and the performance of young male tennis players

We evaluated and compared the total numbers for each group with the total numbers for the top 30 ranked at the end of the year and the total numbers of the bottom 30 ranked at the end of the year to more clearly understand the influence of relative age on the performance of young tennis players.

**Fig 1:**
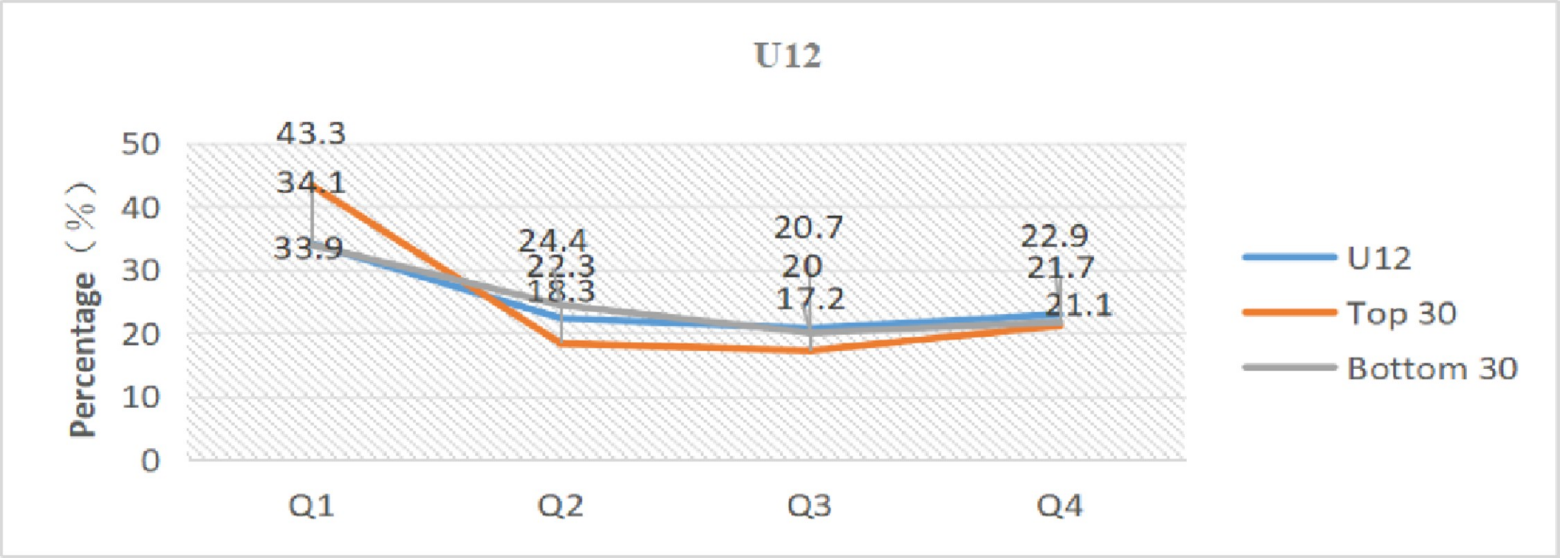
Distribution of birth quartile in the U12 group considering different sport performance ranking.

**Fig 2:**
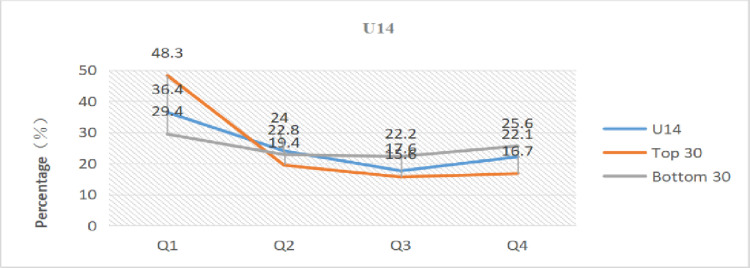
Distribution of birth quartile in the U14 group considering different sport performance ranking.

**Fig 3:**
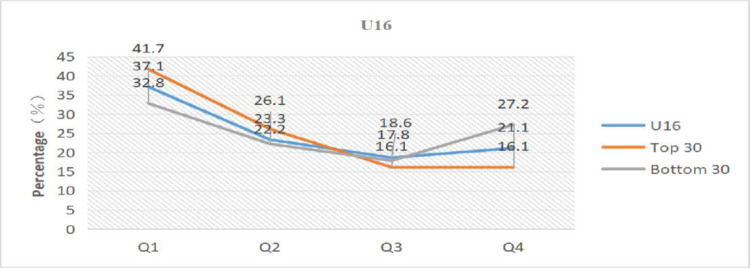
Distribution of birth quartile in the U16 group considering different sport performance ranking.

Table 5 and Figs 1–3 present the results. The percentage of athletes born in the first quarter in the top 30 of all age groups is higher compared to the overall and bottom 30, while the percentage of athletes born in the fourth quarter is lower than the overall and bottom 30. Athletes born in the first quarter even reached 48.3% in the top 30 of U14. In comparison to the bottom 30, the top 30 athletes had birth dates that were 9.4% (U12), 8.9% (U14), and 8.9% (U16) higher in the first quarter and 8.9% (U14) and 11.1% (U16) lower in the fourth quarter, respectively. Specifically, compared to the total, the top 30 athletes’ dates of birth were more heavily weighted in the first quarter than the total for each category: 9.2% (U12), 11.7% (U14), and 4.6% (U16). Conversely, the bottom 30 athletes’ dates of birth were less heavily weighted in the first quarter than the overall total: 0.2% (U12), 7% (U14), and 4.3% (U16).

**Table 5:** Birth quartile distribution, chi-square value, and odds ratio analysis considering the different sports performance ranking from 2014 to 2019.

Group	Classify	Birthdate distribution					Semesters		Data analysis		
		Q1	Q2	Q3	Q4	Total	S1	S2	x²	p	OR
**U12**	n	373	244	227	250	1094	617	477	49.305	.000**	1.49
	%	34.1	22.3	20.7	22.9	100.0	56.4	43.6			
**U12-Top 30**	n	78	33	31	39	180	111	70	32.844	.000**	2.00
	%	43.3	18.3	17.2	21.1	100.0	61.7	38.3			
**U12-Bottom 30**	n	61	44	36	39	180	105	75	8.311	.040*	1.56
	%	33.9	24.4	20.0	21.7	100.0	58.3	41.7			
**U14**	n	313	205	151	190	860	519	341	67.005	.000**	1.66
	%	36.4	24.0	17.6	22.1	100.0	60.4	39.6			
**U14-Top 30**	n	87	35	28	30	180	122	58	52.844	.000**	2.9
	%	48.3	19.4	15.6	16.7	100.0	67.8	32.2			
**U14-Bottom 30**	n	53	41	40	46	180	94	86	2.356	.502	1.15
	%	29.4	22.8	22.2	25.6	100.0	52.2	47.8			
**U16**	n	236	148	118	134	636	384	252	52.553	.000**	1.76
	%	37.1	23.3	18.6	21.1	100.0	60.4	39.6			
**U16-Top 30**	n	75	47	29	29	180	122	58	31.467	.000**	2.59
	%	41.7	26.1	16.1	16.1	100.0	67.8	32.2			
**U16-Bottom 30**	n	59	40	32	49	180	99	81	9.022	.029*	1.20
	%	32.8	22.2	17.8	27.2	100.0	55.0	45.0			

Q1–Q4: the first to the fourth quarter, X²: chi-square value, p > 0.05: no significant difference, p < 0.05: significant difference*, p < 0.01: very significant difference**, OR: ratio of Q1 and Q4.

The skewed distribution of birth dates among the top 30 athletes displayed an extremely significant difference, while the bottom 30 athletes exhibited no difference or a significant difference, according to the data in Table 5. In terms of OR analysis, the OR values of the top 30 athletes were all higher than 2, with the U14-Top 30 group reaching 2.9. The OR values of the bottom 30 athletes were < 1.22 in both the U14 and U16 groups (no effect), with the exception of 1.56 in the U12 group (minimal effect), which was essentially the same as the overall.

Meanwhile, the percentages of the total number in each group whose birth dates were in the first quarter were 34.1% (U12), 36.4% (U14), and 37.1% (U16), and the percentages of people whose birth dates were in the first half of the year were 56.4% (U12), 60.4% (U14), and 60.4% (U16); the ORs were in order: 1.49, 1.66, and 1.76. In general, the degree of birth date skew between the top 30 and bottom 30 athletes varied widely across all groups, with more top-ranked athletes having birth dates closer to the start of the year and more bottom-ranked athletes having birth dates closer to the end of the year. Therefore, athletes with birth dates closer to the start of the year were more likely to achieve greater athletic performance, while those with birth dates closer to the end of the year were less likely to do so.

## Discussion

The key purpose of this study was to examine the RAE among Chinese youth men’s tennis players. The study found that there was a skewed distribution of the birth dates of the athletes in each group. These results are consistent with earlier studies [39, 40, 43–45] that examined the RAE among young tennis players, thus providing continuous evidence for the existence of RAEs in tennis across the world. At the same time, our study revealed the existence of this issue for the first time in Chinese youth tennis and can serve as a starting point for further studies regarding Chinese youth sports as well as future research concerning a means to address it.

There was a significant RAE among all groups of Chinese junior male tennis players, with a skewed distribution of birth dates in each group, and the difference was statistically significant (P < 0.05). The percentages of the total number of athletes in each group whose date of birth was in the first quarter were 34.1% (U12), 36.4% (U14), and 37.1% (U16), and the percentages of athletes whose date of birth was in the first half of the year were 56.4% (U12), 60.4% (U14), and 60.4% (U16); the ORs were 1.49, 1.66, and 1.76, respectively. As the level of play continued to increase, there was a gradual upward trend in the percentage of births in the first quarter and first half of the year from the U12 to the U14 and U16 divisions, and the extent of the impact of the RAE continued to deepen. This trend supports several theoretical models demonstrating the existence of RAE. The Matthew effect is explained by the fact that the rich become richer and the poor become poorer; the Pygmalion effect posits that the greater the expectations of a person, the greater the outcome that person will achieve; and the Galatea effect indicates that once expectations are imposed on a person, that person’s behavior is typically consistent with those expectations [49, 50]. As a result of this pattern, late-born athletes abandon the sport earlier, whereas early-born athletes maintain the advantages they have developed in their early years due to physical considerations and other variables.

The findings mentioned above could be attributed to the selection process used for Chinese youth tennis players as well as the fact that tennis is a sport with numerous youth competitions. Tennis has a more competitive atmosphere due to its early competitive activity and the abundance of youth tournament events throughout the junior years. As a result, those players who were born towards the early end of a particular group are more likely to have superior skills and physical fitness, giving them an advantage over their peers in the same age group [2, 51–53]. Due to the more competitive atmosphere of tennis (as was the case with the junior male tennis players in this study), the same situation was observed among numerous junior athletes. For instance, Dudink et al. [39] discovered that the top 60 tennis players in the Dutch junior league between the ages of 12 and 16 were born in the first three months of the competition year. O’ Donoghue [41] also found that 59.5% of elite junior tennis players were born in the first six months of the year. Agricola et al. [44] found a clear predominance of athletes born in the first half of the year among athletes at the 2007–2011 World Junior Tennis Championships. This dominance extended from junior tennis players to high-level professional athletes, and the presence of the RAE was also still found in both the top 10 WTA ranked athletes [54] and the top ATP ranked athletes [42]. The above results suggest that younger players with tremendous potential are consequently less likely to be selected for elite teams [47] and more likely to withdraw [16]. According to Gould’s research [55], 35% of children in the United States withdraw from organized sports each year, thus squandering a substantial amount of athletic talent.

It is critical that equal opportunities are available to all players. However, many of the procedures that are currently used for selecting athletes do not ensure such fair opportunities. The performance of young players accounts for the majority of the selection criteria for Chinese young tennis players, which undoubtedly increases the probability of this unfair phenomenon. Children who mature later may experience discrimination under the existing selection process. The first group of talented athletes who withdraw from the selection process are frequently those who are temporarily less physically and psychologically mature [51–53]. The second wave of withdrawal typically occurs between the ages of 12 and 14, during the growth spurt period, when many highly competitive sports organizations might eliminate players due to varying rates of biological development. These elements contribute to the increased dropout rate among children who mature later [41–53].

RAE is most plainly present in the Chinese national men’s junior tennis team due to the aforementioned potential causes. The proportion of athletes born in the first half of the year reached as high as 65.4% in the Chinese national junior tennis team’s birth date distribution, which was markedly skewed (P < 0.01, OR 2.8). Therefore, Duarte et al. [56] suggest that the RAE contributes to the exclusion of late-born players from competitive youth football not only because of later physical development but also due to factors such as coaches’ perceptions, behavioral variables, and training environments. In addition, it has been noted in other studies that the introduction of international competitions at the youth elite level has become a crucial component of the elite development system to identify, cultivate, and develop sports talents [57]; it also serves as a mechanism to recognize athletic excellence [58]. Consequently, the aforementioned phenomenon is exacerbated. It is crucial to consider whether youth international competitions increase or diminish participation and development in youth sports [33].

Studies regarding the relationship between the RAE and sports performance in different sports have reached different conclusions. Although studies have demonstrated a relationship between the RAE and performance among young alpine skiers [59], other studies, such as that by Kirkendall [60], have failed to uncover a relationship between the RAE and performance among U-16 players in North American football clubs. Due to the RAE, junior tennis players with birth dates closer to the beginning of the year were more likely to have better sports performance, while those with birth dates closer to the end of the year were less likely to have better sports performance. The higher-ranked athletes were more severely affected by the RAE, whereas the lower ranked athletes were less severely affected, based on the examination of the distribution of birth dates and OR values. This is consistent with the findings of various previous studies. For example, Gutierrez et al.’s study [61] found that the RAE was more prevalent in higher-level sports and more performance-oriented teams.

From the perspective of the sustainable development of sports and the reduction of brain drain, competition organizations, coaches, athletes, and their parents should fully understand the negative factors influencing the relative age effect on the growth and success of athletes. To mitigate the temporary disadvantage of younger players, the talent selection and deselection process should consider and favor player potential development rather than current performance. Some studies have thus suggested that coaches must keep all players to some extent and avoid selecting only the best athletic performers. As a result, late-born players should continue to be involved with the club and be given the same possibilities for playing and training as early-born players [62]. This might diminish the effects of the RAE and increase opportunities for athletes who were born later. Additionally, it has been proposed that late-born athletes must overcome physiological and anthropometric disadvantages in the initial stages of their careers, which may facilitate the development of cognitive and mental abilities that facilitate athletic performance at older ages [63, 64]. To effectively mitigate the impact of the relative age effect, we suggest that China’s youth tennis competition organizations, coaches, players, and parents fully understand the negative impact of the relative age effect on the growth and development of athletes. This can be done by reforming the grouping indices of the youth age-graded tournaments, the selection standards of youth athletes, and other measures. Secondly, junior tennis coaches should consider the RAE’s impact on training, selection, and other factors in addition to physical form and physical quality; they should fully account for the proportion of athletes born in various years and seasons when assembling teams.

Some limitations should be underlined when interpreting this study. Firstly, we have considered the quarterly distribution of birth dates as being equal. The distribution of birth dates for the overall population in the studied nations was not taken into consideration when executing this investigation. Secondly, only quantitatively collected data were used in our study, which slightly limited our ability to fully understand the RAE issue. Ultimately, the study’s findings contributed to a deeper understanding of RAE-related issues among coaches, athletes, and administrators. However, our study did not offer any suggestions regarding how to lessen the effect of the RAE in sports practice.

## Conclusion

The study found that there was a skewed distribution of the athletes’ birth dates in each group. Athletes born in the first quarter were significantly were prevalent than those born in the fourth quarter, and those born in the first half of the year were also significantly more common than those born in the second half of the year. The phenomenon of the RAE exists in all groups of Chinese young men’s tennis players. The RAE has a tendency to gradually strengthen its influence among young Chinese men’s tennis players as they become older.

A deeper impact of the RAE was discovered in the examination of athletes selected for the national junior tennis team and the training reserve team, and in some years, the percentage of players born in the first quarter might reach 50%. This also further suggests that RAE has different degrees of influence on young male tennis players, with a tendency for that influence to increase with the level. In general, the RAE exerts an impact on the athletic performance of athletes participating in Chinese youth tennis competitions. This demonstrates that athletes with birth dates closer to the start of the year have a higher probability of achieving excellent sports performance, whereas athletes with lower rankings have a more difficult time achieving strong outcomes.

## Supporting information

S1 Data (XLSX)
